# Meningeal Lymphatics in Central Nervous System Diseases

**DOI:** 10.1146/annurev-neuro-113023-103045

**Published:** 2024-07-01

**Authors:** Andrea Francesca M. Salvador, Nora Abduljawad, Jonathan Kipnis

**Affiliations:** 1 Brain Immunology and Glia (BIG) Center and Department of Pathology and Immunology, Washington University School of Medicine, St. Louis, Missouri, USA; 2 Neuroscience Graduate Program, Washington University School of Medicine, St. Louis, Missouri, USA

**Keywords:** neuroimmunology, meninges, lymphatic vessels, drainage, central nervous system, neurodegeneration

## Abstract

Since its recent discovery, the meningeal lymphatic system has reshaped our understanding of central nervous system (CNS) fluid exchange, waste clearance, immune cell trafficking, and immune privilege. Meningeal lymphatics have also been demonstrated to functionally modify the outcome of neurological disorders and their responses to treatment, including brain tumors, inflammatory diseases such as multiple sclerosis, CNS injuries, and neurodegenerative disorders such as Alzheimer’s and Parkinson’s diseases. In this review, we discuss recent evidence of the contribution of meningeal lymphatics to neurological diseases, as well as the available experimental methods for manipulating meningeal lymphatics in these conditions. Finally, we also provide a discussion of the pressing questions and challenges in utilizing meningeal lymphatics as a prime target for CNS therapeutic intervention and possibly drug delivery for brain disorders.

## INTRODUCTION: THE MENINGEAL LYMPHATIC SYSTEM

Most organs are supplied by a network of lymphatic vasculature. Lining the lymphatic vessels (LVs) are lymphatic endothelial cells (LECs), which have a distinct identity and transcriptional program compared to blood endothelial cells (Petrova & Koh 2020). LVs function to return filtered interstitial fluid (ISF) from within tissues back to the circulation. Thus, the lymphatic system is essential for maintaining proper tissue fluid balance and waste clearance, with deficits in lymphatic function causing painful swelling and edema. In addition, lymphatics relay this ISF—containing macromolecules, potential antigens, and immune cells—to strategically placed lymph nodes, which are hubs for immune cell activity. As a result, the lymphatic system also critically contributes to tissue immune surveillance and the mobilization of tissue-specific immune responses to challenges.

Although the brain and spinal cord parenchyma lack lymphatic vasculature, a network of LVs exists in the meninges overlying these tissues. The earliest descriptions of brain meningeal lymphatics were made over 200 years ago by the Italian anatomist Paolo Mascagni (Bucchieri et al. 2015, Mascagni 1786, Sandrone et al. 2019). However, these observations were undermined and discounted by the scientific mainstream, which maintained that the central nervous system (CNS) was immune privileged and had no ability to communicate with the peripheral immune system, in part due to the absence of a lymphatic system that would drain the CNS. In 2015, a detailed structural and functional characterization of the meningeal lymphatic system emerged and challenged this dogma (Aspelund et al. 2015, Louveau et al. 2015). Meningeal LVs with distinct physiological properties are found in the dorsal aspect and basal parts of the skull (Ahn et al. 2019), track along venous sinuses of the dura, and functionally connect with deep cervical lymph nodes (dCLNs) (Jacob et al. 2022, Louveau et al. 2015). They express bona fide lymphatic markers such as prospero homeobox protein 1 (PROX1), vascular endothelial growth factor receptor 3 (VEGFR3), lymphatic vessel endothelial hyaluronan receptor 1 (LYVE1), podoplanin, and CC motif chemokine ligand 21 (CCL21) (Aspelund et al. 2015, Louveau et al. 2015). In the dorsal meninges, initial lymphatics are located along the rostral rhinal vein, superior sagittal sinus, transverse sinus, confluence of sinuses, pterygopalatine artery, and middle meningeal artery (das Neves et al. 2021). Lymphatics also line the petrosquamosal and sigmoid sinuses, where they display morphologies reminiscent of collecting lymphatics. Meningeal lymphatics are also present in the skull base, where they are equipped with zipper-type junctions and lymphatic valves (Ahn et al. 2019).

Murine meningeal LVs begin their development postnatally, in contrast with other peripheral lymphatic networks that appear during embryogenesis (Antila et al. 2017, Izen et al. 2018). Meningeal lymphatics are first observed at the base of the skull, extending medially and dorsally to achieve full maturation by postnatal day 28 (Antila et al. 2017, Izen et al. 2018). Vascular endothelial growth factor C (VEGFC), possibly secreted by meningeal smooth muscle cells, is critical for the development and maintenance of meningeal lymphatics (Antila et al. 2017). VEGFC signals directly onto LECs through the receptor VEGFR3, and this requirement for VEGFC-VEGFR3 informed the design of several experimental methods for manipulating meningeal lymphatic function. These and other approaches are described in detail in the Supplemental Appendix and Supplemental Table 1. However, the cellular precursor(s) for meningeal LECs has not been identified. While development in proximity to blood vessels may suggest a blood endothelial source, accumulating studies have demonstrated heterogeneous, organ-specific origins for LECs. In addition, how meningeal lymphatics are replenished is another matter of active investigation. Existing lymphatics may undergo sprouting in disease contexts (Bolte et al. 2020). Meningeal lymphatics that regress upon disruption of VEGFR3 signaling can recover upon removal of the pharmacological inhibitor (Antila et al. 2017). Additionally, skull progenitor cells have been recently shown to also contribute to VEGFC secretion and meningeal lymphatic cell growth (Ma et al. 2023).

Functionally, meningeal lymphatics are now appreciated to drain cerebrospinal fluid (CSF), along with CNS waste products and antigens, from the brain to cervical lymph nodes (CLNs) (Da Mesquita et al. 2018b; Louveau et al. 2015, 2018). Despite their segregation from the brain parenchyma, meningeal lymphatics also influence the influx and efflux of CSF macromolecules in the parenchyma through a paravascular or glymphatic pathway (Da Mesquita et al. 2018b). It must be noted that there are reports suggesting the detection of lymphatics in the leptomeninges (Shibata-Germanos et al. 2020), pia mater (Cai et al. 2019), and more recently, subarachnoid lymphatic-like membrane that divides the subarachnoid space into two compartments (Møllgård et al. 2023). However, the prevailing consensus is that the meningeal lymphatics are restricted to the dura mater (das Neves et al. 2021).

**Figure 1:**
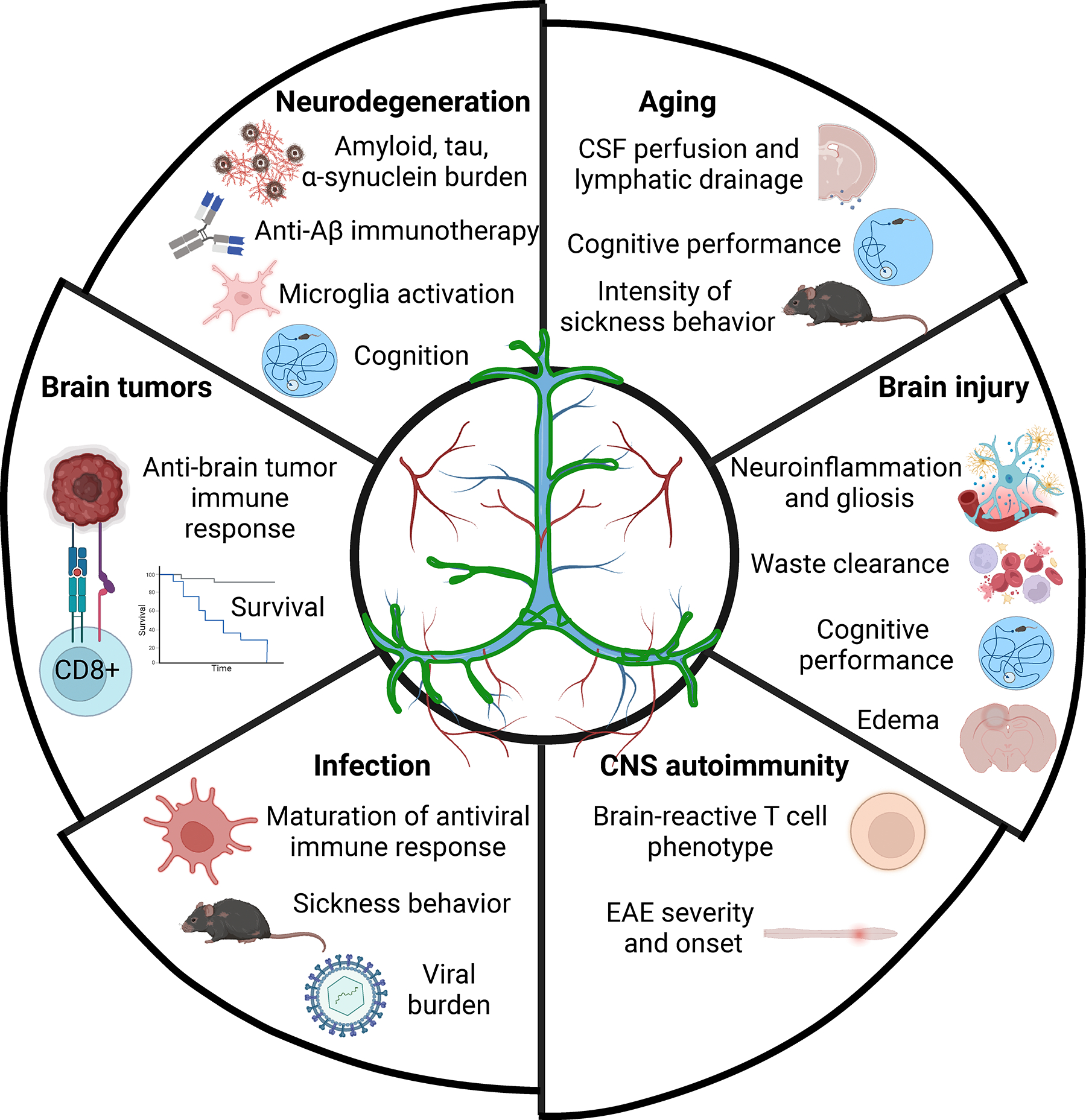
Aspects of neurological diseases affected by meningeal lymphatic vessels. Summary of neurological diseases discussed in this review and the multiple facets regulated by meningeal lymphatic (dys)function. Figure adapted from images created with Biorender.com.

Notably, while these findings were made in mice, meningeal lymphatics have also been documented in other organisms. For example, the optical clarity of zebrafish has enabled real-time imaging of intracranial lymphatic sprouting and the trafficking of immune cells (Castranova et al. 2021). In human and nonhuman primates, high-resolution magnetic resonance imaging (MRI) was used to noninvasively describe a meningeal lymphatic network with three-dimensional anatomy that was highly similar to rodents (Absinta et al. 2017, Jacob et al. 2022). The discovery of an evolutionarily conserved meningeal lymphatic system sheds light on a new route for CSF efflux and demonstrates a direct link between the CNS and peripheral immunity, which has been pivotal to broadening our understanding of the pathophysiology of CNS disorders (Figure 1).

## NEURODEGENERATION: ALZHEIMER’S DISEASE

Among the hallmarks of age-related neurodegenerative disorders such as Alzheimer’s disease (AD) and Parkinson’s disease (PD) is the inadequate clearance of pathological protein aggregates that eventually accumulate in excess, leading to neuroinflammation and progressive neuronal loss (Tarasoff-Conway et al. 2015). There are multiple, overlapping clearance systems that help maintain CNS health (Tarasoff-Conway et al. 2015), and the contribution of the meningeal lymphatic system to this process is becoming increasingly recognized.

The involvement of lymphatic drainage in AD was noted in studies demonstrating amyloid-β (Aβ) accumulation in anatomical locations that are accessible to lymphatics such as CSF (Bateman et al. 2012), dura mater (Joachim et al. 1988), and CLNs of AD patients and murine models (Pappolla et al. 2014). Although the structure and function of meningeal lymphatics appear normal in young murine models of amyloidosis (i.e., J20, 5xFAD) (Da Mesquita et al. 2018b), impairments in meningeal lymphatics have been observed in aged 5xFAD mice. This finding correlated with Aβ deposition around meningeal blood and lymphatic vasculature (Da Mesquita et al. 2021b, Kwon et al. 2019). Alterations in meningeal lymphatic function in older AD animals might be due to the synergism of aging and Aβ toxicity (das Neves et al. 2021). In support of a toxic effect of Aβ on LECs, bulk RNA sequencing of LECs from 6-month-old 5XFAD mice, which exhibit amyloid presentation as early as 2 months old (Oakley et al. 2006), revealed significant changes in the expression of genes related to exocytosis and response to low-density lipoprotein (Da Mesquita et al. 2021b) compared to age-matched wild-type controls.

Meningeal lymphatic dysfunction is present in aged animals (Da Mesquita et al. 2018b), and inducing similar dysfunction in young transgenic AD mice through extended photodynamic ablation or surgical ligation exacerbates Aβ-related pathology in the brain and meninges (Da Mesquita et al. 2018b, Wang et al. 2019). Notably, meningeal lymphatic ablation leads to a remarkable meningeal accumulation of Aβ together with increased macrophage recruitment, which was also observed in the dura mater of human AD patients (Da Mesquita et al. 2018b). Impairing meningeal lymphatics in young transgenic AD mice also leads to worsened cognitive function, microglia reactivity, and dysfunctional vascular transcriptomic signatures (Da Mesquita et al. 2021b). Interestingly, microglial transcriptional signatures from AD mice with meningeal lymphatic dysfunction overlap with those of human microglia from AD patients (Da Mesquita et al. 2021b). These findings suggest that meningeal lymphatics shape CNS innate immunity and form a critical aspect of human AD pathogenesis.

Therapeutic enhancement of meningeal lymphatics through intracisternal VEGFC delivery augments lymphatic drainage, glymphatic flow, and cognition in aged mice (Da Mesquita et al. 2018b). In the context of AD, administration of recombinant VEGFC protein to 9-month-old APP/PS1 mice also enhanced lymphangiogenesis, reduced amyloid deposition, and improved cognitive performance (Wen et al. 2018). However, viral-mediated VEGFC treatment of 7-month-old J20 mice did not alter AD-related pathology (Da Mesquita et al. 2021b). The different outcomes may be due to differences in plaque development between mouse strains or the VEGFC treatment regimen. Intact meningeal lymphatics are also critical for the efficiency of passive immunotherapy (Sevigny et al. 2016) against Aβ (Da Mesquita et al. 2021b). Young mice receiving monoclonal antibodies against Aβ had reduced plaque burden. The beneficial effects were not observed in aged AD animals, perhaps due to limited antibody influx to the parenchyma, but this could be improved with VEGFC treatment (Da Mesquita et al. 2018b, 2021b). Aside from VEGFC, Down syndrome critical region gene 1 (*Dscr1*) upregulated during lymphangiogenesis may potentiate meningeal lymphatics (Choi et al. 2021). Transgenic mice overexpressing DSCR1 in a 5xFAD background (DSCR1/5xFAD) or ectopic expression of DSCR1 in 5xFAD mice reduces amyloid deposition, improves lymphatic drainage, and enhances cognitive performance, but ligation abolishes these beneficial effects.

Aside from amyloid deposition, AD is also marked by the presence of intracellular neurofibrillary tangles propagating in the brain in part through Aβ plaques (Götz et al. 2001, Hurtado et al. 2010). Meningeal lymphatics have not been as extensively studied in tau models, but the increased Aβ burden observed with meningeal lymphatic ablation may denote its relevance. Nevertheless, it has been reported that monomeric tau injected into brains of K14-VEGFR3-Ig mice lacking dural lymphatics had greater brain retention and delayed extracellular clearance compared to controls (Patel et al. 2019). Targeted ablation of meningeal lymphatics or enhancement of its function in tau transgenic animals has yet to be performed, but it will help resolve confounding factors such as abnormal patterning of other lymphatic beds and lymphedema reported in adult K14-VEGFR3-Ig mice (Mäkinen et al. 2001).

Future investigation of how manipulating meningeal lymphatic function affects the immune response in AD models, which may modulate disease progression (Chen et al. 2023, Ennerfelt et al. 2022, Gratuze et al. 2021, Wang et al. 2022), may extend our understanding of the contribution of meningeal lymphatics to AD pathology and inform the development of novel therapies against neurodegeneration.

## NEURODEGENERATION: PARKINSON’S DISEASE

PD is marked by the progressive loss of dopaminergic neurons and the buildup of misfolded forms of α-synuclein (Lo Bianco et al. 2002). Alterations of meningeal lymphatics and lymphatic drainage have been described in animal models of PD, as well as in PD patients (Ding et al. 2021). Transgenic A53T mice, overexpressing a mutated form of human α-synuclein (Giasson et al. 2002), have enlarged dCLNs and reduced tracer perfusion to the dCLNs (Liu et al. 2023), which may be attributed to increased macrophage activation and inflammation in the dCLNs. Intrastriatal injection of α-synuclein preformed fibrils (PFFs), which may cause α-synuclein toxicity (das Neves et al. 2021), impaired lymphatic drainage of CSF-injected tracer and reduced tight junction protein expression in meningeal LECs (Ding et al. 2021). This impairment also correlated with an increase in macrophage accumulation and proinflammatory cytokine expression in the meninges. Idiopathic Parkinson’s disease (iPD) patients scanned with MRI similarly presented with reduced lymphatic outflow and dCLN drainage despite the absence of significant morphological changes in meningeal LVs (Ding et al. 2021). The clinical data may form the basis for diagnostic strategies for the early stages of iPD. Collectively, these findings suggest that α-synuclein may impair meningeal lymphatic function, and prolonged meningeal lymphatic dysfunction may also exacerbate inflammation.

Impairing meningeal lymphatic drainage can also exacerbate PD pathology, as ligation of dCLN afferents in A53T mice worsened α-synuclein aggregation in the brain, hinting at glymphatic dysfunction (Zou et al. 2019). The elevation of α-synuclein in the brain of ligated A53T mice was also accompanied by increased gliosis, proinflammatory cytokine expression, and tau levels, evidencing a cross talk of proteinopathies. Both dopaminergic neuron loss and motor dysfunction were accelerated upon lymphatic drainage impairment. Prior meningeal lymphatic dysfunction in mice receiving intrastriatal injections of α-synuclein PFFs also resulted in significantly more insoluble aggregate accumulation in the brain and worsened motor and sensory function (Ding et al. 2021).

## CNS AUTOIMMUNITY

Multiple sclerosis (MS) is a debilitating neuroinflammatory disorder characterized by demyelinating lesions and dramatic immune cell infiltration into the CNS (Dendrou et al. 2015). While its etiology remains unresolved, it is widely considered an autoimmune disease and is commonly modeled by immunizing mice with myelin antigens to produce experimental autoimmune encephalomyelitis (EAE) (Stromnes & Goverman 2006). Because meningeal lymphatics sit at the interface between the CNS and the peripheral immune system, they can influence the drainage of tissue antigens and antigen-presenting cells (APCs) from the CNS to draining lymph nodes to modify disease progression.

Peripheral lymphatics may proliferate and expand during inflammation (Kim et al. 2014). Upregulation of VEGFC has been reported in the CNS of rodents with EAE (Hsu et al. 2019, Park et al. 2013). Curiously, lymphatics in the cranial and spinal cord dura did not display significant morphological changes when assessed at multiple time points during EAE (Hsu et al. 2019, Louveau et al. 2018). Lymphatics in the nasal cavity, however, expanded at later time points during EAE (Louveau et al. 2018) and in mice that have a severe EAE score (Hsu et al. 2019), which may be through the proliferation of existing lymphatics. The differential response of meningeal and nasal lymphatics during EAE may be attributed to the intrinsic heterogeneity of lymphatic beds or the relative availability of growth factors. It is also possible that meningeal lymphatics in EAE are instead altered transcriptionally or functionally, as lymphatics undergo changes in drainage performance during inflammation (Schwager & Detmar 2019).

While meningeal lymphatic structure appears unchanged in EAE, meningeal lymphatics contribute functionally to disease development. Meningeal lymphatic impairment using local photodynamic ablation or ligation of dCLN afferents delayed the onset and attenuated the severity of EAE compared to controls (Louveau et al. 2018). Photodynamic ablation of nasal lymphatics or ligation of afferents to the brachial lymph nodes, on the other hand, altered neither disease progression nor T cell accumulation in the dCLNs. These findings suggest that the ability of meningeal lymphatics to modify disease may involve their connection to the dCLNs where antigens, APCs, and T cells are drained, and T cells are potentially licensed to become encephalitogenic (Louveau et al. 2018, Rustenhoven & Kipnis 2022). Reports demonstrating the presence of brain antigens in the CLNs of MS patients or EAE animal models support this, or they may indicate the extent of CNS damage (van Zwam et al. 2009b). Additionally, dCLN excision delayed disease onset and reduced disease severity, but it was not sufficient to abolish EAE (Furtado et al. 2008, Louveau et al. 2018, van Zwam et al. 2009a). This finding indicates that other drainage routes are tapped in the absence of meningeal lymphatics or dCLNs to establish disease. Understanding how immune cells interact with meningeal lymphatics or the specific reaction occurring in the dCLNs to promote EAE may be therapeutically relevant. Meningeal lymphatics may also control CSF influx to the adjacent skull bone marrow modulating the supply of myeloid cells to the inflamed CNS (Cugurra et al. 2021, Mazzitelli et al. 2022).

Inhibition of the VEGFC/VEGFR3 pathway, necessary for the maintenance of meningeal lymphatics in adulthood, has been employed to test the function of meningeal lymphatics in EAE (Antila et al. 2017). These results must be interpreted with caution as VEGFR3 may also be expressed in blood endothelial cells (Da Mesquita et al. 2021b, Rustenhoven et al. 2021). Nevertheless, intraperitoneal administration of a VEGFR3 tyrosine kinase inhibitor, MAZ51, prior to symptom onset but after immunization, delayed and attenuated the EAE disease score (Hsu et al. 2019). This treatment led to the regression of dorsal meningeal and nasal lymphatics, indicating that the therapeutic benefit of MAZ51 involves either or both lymphatic structures. However, other recent studies report that disrupting VEGFR3 signaling before immunization using (*a*) systemic administration of VEGFR3-blocking antibodies or AAV-VEGFC/D trap and (*b*) conditional deletion of VEGFR3 in Prox1^+^ cells during adulthood did not alter the development of active EAE despite the regression and functional impairment of dorsal meningeal lymphatics (Li et al. 2023). Additionally, ablating meningeal lymphatics through intracisternal delivery of AAV-VEGFC/D did not alter the disease course nor the trafficking of pathogenic T cells in an adoptive transfer model of EAE (Merlini et al. 2022). The conflicting outcomes may be due to differences in the method (i.e., systemic versus local; genetic, surgical, or pharmacological) and timing of manipulation. A thorough characterization of potential unintended effects of meningeal ablation experiments will help clarify the contribution of meningeal lymphatics to CNS autoimmune disorders and will aid in developing strategies that change the onset or progression of the disease, while providing a more localized VEGFC supply may help to determine how boosting meningeal lymphatic function affects neuroinflammation.

## CNS TUMORS

Recent works have identified a critical role for meningeal lymphatics in antitumor immunity. Photodynamic ablation of dorsal meningeal lymphatics prevents dCLN lymphangiogenesis and enlargement that are typically observed after glioma injection to the brain (Hu et al. 2020), suggesting a dampened peripheral immune response in the absence of meningeal lymphatics. This observation was accompanied by cerebral edema and a reduction in tracer drainage from the tumor to dCLNs, implicating the drainage functions of meningeal lymphatics. Conversely, prophylactic VEGFC treatment led to near-complete, long-lasting glioma rejection in mice, dependent on drainage to the dCLNs and T cell function (Song et al. 2020). VEGFC treatment also increased the priming and clonal expansion of tumor antigen-specific T cells in extracranial lymph nodes (Song et al. 2020). Together, these experiments provide strong evidence that meningeal lymphatic drainage of tumor antigens is necessary for mobilizing peripheral immunity against gliomas.

In line with this, stimulating meningeal lymphatic function has emerged as a promising strategy for boosting the efficacy of glioma immunotherapy. Mice injected with glioma cells engineered to overexpress VEGFC showed longer survival and smaller tumor volumes in response to immune checkpoint inhibition compared to mice bearing control gliomas (Hu et al. 2020). This improvement was accompanied by an increase in proliferative CD8^+^ T cells and a reduction in regulatory T cells in the tumor and dCLNs. Importantly, meningeal lymphatic ablation or treatment with anti-CCL21/chemokine receptor 7 (CCR7) antibodies abolished the therapeutic benefit of VEGFC, further confirming the role of meningeal lymphatics in immune cell drainage. In a companion study, VEGFC messenger RNA (mRNA) was used to improve the efficacy of checkpoint inhibitors in two models of mouse glioma, and the effect was dependent on T cell function (Song et al. 2020). When combined with anti-PD1, VEGFC mRNA also conferred long-term resistance to glioma, with T cells from surviving mice providing protection to naïve mice. In these studies, while VEGFC mRNA was taken up by a variety of cells in the brain, meninges, and dCLNs, VEGFC protein was confined to the CSF and meninges, and VEGFR signaling was active only in LECs (Song et al. 2020). Lastly, in addition to immune checkpoint inhibitors, VEGFC also boosted the efficacy of radiation therapy in mouse glioma models via increased drainage of dendritic cells (DCs) from the tumor to the dCLNs (Zhou et al. 2022). These preclinical studies highlight the potential of meningeal lymphatic-based therapeutic approaches for the treatment of brain tumors.

It should be noted that LVs have classically been thought to contribute to tumor cell dissemination leading to metastasis (Oliver et al. 2020). In glioblastoma (GBM) patients, the clinical occurrence of extraneural metastasis is relatively rare and may correlate with craniotomy or radiotherapy (Goodwin et al. 2016). In the studies discussed above, VEGFC mRNA was not found to promote the metastasis of glioma cells to the dCLNs (Song et al. 2020). Glioma cells injected directly into the CSF drain to dCLNs, and Visudyne-based ablation of dorsal meningeal lymphatics reduced this drainage (Hu et al. 2020). However, it is unclear whether tumor cells clustered in the brain parenchyma gain access to CSF, or if tumor cells that reach the periphery do so via other routes. Future studies should continue to explore these possibilities.

Other studies have characterized the effect of glioma on meningeal lymphatic function. Human GBM tissue exhibits a reduction of VEGFC (Song et al. 2020), limiting lymphangiogenic signals. Despite this, meningeal lymphangiogenesis was observed in mice 2 weeks after receiving intrastriatal glioma injection (Hu et al. 2020). Lineage tracing using *Prox1-CreER*^*T2*^;*tdTomato* mice revealed that meningeal lymphangiogenesis after glioma cell injection was attributed to the sprouting of existing vessels. However, a different study demonstrated reduced CSF drainage to the dCLNs and superficial CLNs 14 days after striatal glioma injection (Ma et al. 2019b). These experiments showed an accumulation of tracer in the cisterna magna of glioma mice and reduced CSF perfusion of the brain. It is possible that the meningeal lymphangiogenesis observed may be insufficient to restore healthy levels of drainage. Reductions of CSF flow in glioma could have deleterious consequences for antitumor immunity, intrathecal drug delivery, edema, or waste buildup.

Meningeal lymphatics may also play a role in the progression of meningiomas, the most common primary intracranial tumor (Ostrom et al. 2022). A recent comprehensive multiomics study stratified meningiomas into three clinical subtypes: Merlin/Nf2 intact, immune enriched, and hypermitotic (Choudhury et al. 2022). The immune-enriched subtype with moderate clinical outcome was first characterized by human leukocyte antigen (HLA) induction, but it was also distinguished by a histological increase in LVs, hypomethylation of lymphatic markers *Lyve1* and *Ccl21*, and increased expression of extracellular matrix remodeling genes linked to lymphangiogenesis. These findings suggest that meningeal lymphatics may be relevant to this meningioma subtype and targeting them might be a novel therapeutic strategy for combatting the disease.

## BRAIN INJURY

Brain injuries caused by mechanical trauma or stroke profoundly alter the CNS environment (Salvador & Kipnis 2022). Injury leads to cell death and the release of debris that must efficiently be cleared (Bolte et al. 2020), and it triggers a complex immune response involving innate and adaptive effectors (Iadecola et al. 2020). Meningeal lymphatics are central to these processes as they facilitate communication between the brain and its surrounding compartments.

Studies have investigated the impact of brain injury on the structure and function of meningeal lymphatics. Lymphangiogenesis near the sagittal sinus was reported two weeks after photothrombotic ischemic stroke (Yanev et al. 2020) likely due to direct lymphatic damage. In support of this theory, the absence of lymphangiogenesis was reported in transient middle cerebral artery occlusion, a different ischemic stroke model (Yanev et al. 2020). Lymphangiogenesis observed 2 weeks after hemorrhagic stroke (Tsai et al. 2022, Virenque et al. 2021) correlated with improved lymphatic drainage. Interestingly, improved bead drainage was also observed in a model of subarachnoid hemorrhage without detectable lymphangiogenesis (Chen et al. 2020). In contrast, deficits in the drainage of beads delivered to the CSF were detected 2 h after mild closed-skull traumatic brain injury (TBI). The impairment persisted up to 1 month, accompanied by morphological changes in the dorsal meningeal lymphatics. This finding suggests that TBI leads to meningeal lymphatic dysfunction, and this was similarly observed in a hydraulic shock model of TBI in rats (Liao et al. 2023). Induction of subdural hematoma in rodents also led to meningeal lymphatic drainage defects and downregulation of lymphatic marker expression (Liu et al. 2020). Given these findings, it is pivotal to determine the factors that alter meningeal function following brain injury. Unlike stroke, mechanical trauma might directly injure LVs, but it is also possible that changes in intracranial pressure (ICP) (Haider et al. 2018) are sensed by LVs, causing them to remodel or become dysregulated. In agreement with this idea, increasing ICP through bilateral internal jugular vein ligation resulted in an acute reduction in the drainage of fluorescently labeled intracisternal-injected beads to the meninges and the dCLNs (Bolte et al. 2020). Activation of the mechanosensory channel Piezo1 may contribute to lymphatic sprouting (Choi et al. 2017, 2022). A similar mechanism may underlie meningeal LV remodeling after injury, which warrants further investigation as Piezo1 agonists, such as Yoda1, have been suggested to ameliorate lymphedema (Choi et al. 2022) and conditions with increased ICP such as craniosynostosis and aging (Matrongolo et al. 2024).

Meningeal lymphatics may also play a role in alleviating brain edema. Edema disrupts ICP, causes brain herniation, and exacerbates cell death due to ion imbalance and ATP depletion (Perla et al. 2023). Acute poststroke edema was shown to result from the influx of excess CSF along enlarged perivascular spaces (Mestre et al. 2020), which overturned the prevailing model of brain edema stating that blood influx initiates acute tissue swelling after stroke. Additionally, preserving lymphatic function through the intracisterna magna delivery of VEGFC or 9-*cis*-retinoic acid, another lymphangiogenic factor, attenuated brain edema, oxidative stress, and neuroinflammation after TBI (Liao et al. 2023). Prior meningeal LV dysfunction induced by the ligation of the afferent LVs to the dCLNs, photoablation of meningeal LVs, or aging also exacerbated cognitive performance and inflammation after TBI (Bolte et al. 2020), while improving the function of meningeal LVs in aged mice using viral-mediated VEGFC overexpression reduced Iba1^+^ gliosis after TBI (Bolte et al. 2020). These findings support the critical role of meningeal lymphatics in brain fluid clearance, proper CSF homeostasis, and glymphatic function in the management of brain injuries.

Given the established waste drainage function of meningeal lymphatics, other studies have investigated its role in debris clearance. Fluorescently labeled erythrocytes injected into the CSF were taken up by meningeal lymphatics and drained to CLNs (Chen et al. 2020), showing that meningeal lymphatics can transport blood cells in the CSF. Live imaging of *Prox1* reporter mice injected with intravascular tracers showed uptake of blood contents by meningeal LVs after intracerebral hemorrhage, peaking 1 h after injury (Virenque et al. 2021). Notably, focal laser rupture of dural vasculature did not result in meningeal lymphatic uptake of intravascular tracer, suggesting alternative clearance mechanisms. Supporting the role of meningeal lymphatics in hematoma clearance, disrupting meningeal lymphatic function worsened acute intracerebral hematoma volume, prevented subarachnoid clot clearance, and reduced subarachnoid erythrocyte drainage to dCLNs (Tsai et al. 2022). Meanwhile, pretreatment with VEGFC reduced intracerebral hematoma volume (Tsai et al. 2022).

Meningeal lymphatics may also contribute to the acute, innate response to injury by regulating proper CSF flow. Recent studies have demonstrated that CSF carries CNS-derived injury signals to the overlying skull bone marrow, which initiates myelopoiesis to directly send neutrophils to the CNS through vascular channels in the skull (Courties et al. 2015, Herisson et al. 2018, Mazzitelli et al. 2022, Pulous et al. 2022). Given that neutrophils are the first peripheral immune cells to infiltrate the brain after injury (Iadecola et al. 2020, Salvador & Kipnis 2022), proper CSF perfusion is critical for the initiation of a rapid injury response.

Brain-derived antigens such as myelin basin protein (MBP), myelin oligodendrocyte glycoprotein (MOG), microtubule-associated protein 2 (MAP2), neurofilament light polypeptide (NF-L) and NMDA receptor subunit (NR-2A) have been observed in the CLNs of both mice and humans after stroke (Planas et al. 2012). This drainage may result in antigen-specific responses or reflect the initial tissue damage. In stroke patients, MBP immunoreactivity in the CLNs correlated with larger infarct volumes and poor clinical outcomes, while MAP2 immunoreactivity correlated with smaller stroke volumes and better functional recovery (Planas et al. 2012). In addition, surgical removal of CLNs prior to ischemia induction in mice altered infarct size (Esposito et al. 2019). Likely downstream of antigen drainage, T cells accumulating in the CNS after stroke regulate infarct size in a cell-specific manner. Regulatory T cells limit infarct size (Ito et al. 2019), while gut-derived, IL-17^+^ γδ T cells increase tissue damage after ischemia in mice (Benakis et al. 2016). Notably, these populations appear in the brain and meninges, respectively, 14 days after stroke, thus constituting long-term responders. The integrity of meningeal lymphatics also alters immune cell infiltration to the brain following controlled cortical impact (CCI) (Wojciechowski et al. 2020). Inducing CCI injury to K14-VEGFR3-Ig mice lacking peripheral and meningeal lymphatics led to a reduction of T cells in perilesional areas 30 days after injury without altering lesion volume. Together, these studies underscore the functional relevance of antigen drainage, T cell trafficking, and CSF flow in brain injuries and imply a role for meningeal lymphatics in their pathogenesis.

## MIGRAINE

Migraines are a leading cause of adult disability (Steiner et al. 2020). While the brain itself does not contain nociceptors, the meninges are innervated by nociceptor fibers emanating from the trigeminal nerve (Levy & Moskowitz 2023) and terminating on meningeal collagen bundles, blood vessels, and lymphatics. Notably, the calvaria is also innervated by nociceptors, and their activation might trigger migraines (Kosaras et al. 2009). Overactivation and sensitization of meningeal nociceptors is thought to underlie migraine pain (Levy & Moskowitz 2023).

Nociceptors are highly responsive to inflammatory stimuli, including ATP, cytokines such as IL-1β and tumor necrosis factor α (TNFα), serotonin, and histamines released from degranulated mast cells that are present near meningeal nerve fibers (Levy et al. 2012; Zhang et al. 2007, 2011, 2012; Zhao et al. 2023). Stimulated nociceptors then produce neurogenic inflammation (Chiu et al. 2012) by secreting neuromodulators, such as calcitonin gene–related peptide (CGRP) and substance P, that recruit leukocytes via increased vasodilation and direct signaling on immune cells. The activated meningeal nociceptors are sensitized to mechanical stimuli, which may trigger or exacerbate migraine in response to otherwise innocuous stimuli such as subtle ICP changes, head movements, or vessel dilation (Strassman et al. 1996). Links between meningeal immunity and migraine are also consistent with the clinical co-occurrence of migraines with inflammatory disorders such as asthma and allergy where mast cells are activated (Levy & Moskowitz 2023, Martin et al. 2016).

The role of meningeal lymphatics in migraine pathogenesis, however, remains understudied. A recent study observed a trend toward increased meningeal mast cell density in mice with genetically ablated LVs (Mikhailov et al. 2022). This finding suggests a possible role for meningeal lymphatics in regulating local immune populations that are available to potentiate or respond to nociceptor activity during migraine pathogenesis. In line with this hypothesis, previous studies have demonstrated T cell accumulation in the meninges of mice with dysfunctional meningeal lymphatics, attributed to a lack of drainage to extracranial lymph nodes (Louveau et al. 2018). Manipulating the meningeal immune landscape via changes in lymphatic drainage may shape the neuroimmune interactions underlying migraine pain.

Recent work has also pointed toward a role for brain ISF efflux and CSF flow in migraine pathogenesis, highlighting the relevance of lymphatic function. Migraines with aura originate from aberrant cortical activity or cortical spreading depolarizations (CSDs) that spread throughout the brain (Bolay et al. 2002, Moskowitz et al. 1993). CSDs lead to cellular stress and the secretion of proinflammatory mediators, such as high mobility group box 1 protein (HMGB1) and IL-1B, into the CSF (Karatas et al. 2013). CSDs also elevate nuclear factor κB in pial astrocytes, which subsequently release vasodilators and proinflammatory cytokines into the subarachnoid space. These algesic and inflammatory mediators may travel through the CSF and subarachnoid space to reach nociceptors and immune populations in the meninges and calvaria, contributing to persistent nociceptor activity (Karatas et al. 2013). Recently, positron emission tomography imaging revealed the presence of inflammatory signals in the meninges and calvaria overlying the occipital lobe of patients with migraine and visual aura (Hadjikhani et al. 2020), paralleling the CSD-induced migration of inflammatory mediators from the brain to dura. Additionally, the role of sleep, which enhances glymphatic function (Xie et al. 2013), in migraines has been examined as acute sleep deprivation increases the frequency and reduces the threshold for CSDs (Negro et al. 2020). Together, these findings suggest that CNS fluid flow may play a key role in migraine progression by allowing cross talk between the brain, meninges, and skull. Future studies should examine whether changes in meningeal lymphatic function may affect migraine progression through changes in CSF clearance.

While early electrostructural studies identified nerve endings on meningeal vasculature, including lymphatics (Andres et al. 1987, Messlinger et al. 1993), the effects of nociceptor activation on lymphatic function have not been determined. Prolonged activation of sensitized nociceptors during migraine might affect meningeal lymphatic function, either directly or via immune mediators, to aggravate pathology. Future studies should examine whether nociceptor-LEC communication contributes to the genesis or exacerbation of migraine pain.

## INFECTION AND SICKNESS BEHAVIOR

Meningeal lymphatics have been reported to influence the development of the immune response to CNS-invading pathogens. In the chronic phase of *Toxoplasma gondii* infection, a robust T cell expansion was observed in the dCLNs correlating with increased parasite burden in the brain (Kovacs et al. 2022). DCs from the dural meninges likely sample the parasite-bearing CSF to prime T cells, which is corroborated by DC accumulation in the dura and dCLNs. Disrupting meningeal lymphatic drainage reduced DC maturation and subsequent T cell activation in the dCLNs, but it did not affect the parasite-specific responses in the brain (Kovacs et al. 2022). This indicates that the immune reaction against the parasite occurring in other lymph nodes is sufficient for neuroprotection. On the other hand, neurotropic viruses that disseminate to the CNS require intact meningeal lymphatic function for host survival (Li et al. 2022). Prior meningeal lymphatic dysfunction induced by photodynamic ablation or surgical ligation of dCLN afferents increased the mortality and viral burden in the brains of mice peripherally infected with neurotropic Japanese encephalitis virus (JEV) (Li et al. 2022), which was accompanied by an increase in proinflammatory cytokine expression and immune cell numbers in the brain. Prophylactic treatment with VEGFC using a slow-release hydrogel extended survival and reduced the viral burden in infected animals. Meningeal lymphatics may thus serve as channels promoting viral elimination through the efficient priming of immune responses in the draining lymph nodes, but it is also possible that their ablation alters the meningeal immune landscape responsible for pathogen clearance (Da Mesquita et al. 2021a, Louveau et al. 2018).

Remarkably, neurotropic viral infection (e.g., JEV, vesicular stomatitis virus, herpes simplex virus 1, rabies, Zika virus) can also promote meningeal lymphangiogenesis, most prominently in the confluence of sinuses, as well as the upregulation of VEGFC in the brain (Li et al. 2022). Despite the expansion of meningeal LVs, JEV intravenous infection impaired the drainage of tracers injected into the CSF and the formation of lymphatic valves. Therefore, the altered lymphatic morphology may indicate compensatory lymphatic hyperplasia related to increased immune cell infiltration (Ahn et al. 2019, Gousopoulos et al. 2016). Understanding the mechanisms contributing to meningeal LV dysfunction during CNS viral infection may enable us to define mechanisms that preserve their integrity in other neurological diseases.

Infection also leads to multifaceted physiological and behavioral changes that are critical for an organism’s survival (Dantzer et al. 2008). While the inflammatory mediators during infection have been described, the contribution of various relay routes between the periphery and the brain remains unclear. The meningeal lymphatic system’s unique anatomical location enables it to alter the delivery and clearance of inflammatory signals in the brain, thereby regulating behavior. Recent work demonstrated that photodynamic ablation of meningeal LVs in adult mice treated with an intraperitoneal injection of IL-1β, a potent inducer of sickness behavior (Aubert et al. 1995), worsened exploratory activity compared to animals with intact meningeal lymphatics (Goldman et al. 2022). Moreover, the exacerbated sickness behavior induced by meningeal lymphatic ablation appears to be mediated by altered microglial activation after IL-1β injection. A similar axis was described in models of AD (Da Mesquita et al. 2021b), indicating that lymphatic drainage is involved in modulating microglial state to regulate behavior.

Aging also leads to exacerbated sickness-related symptoms (Godbout et al. 2005) and, notably, reduced meningeal lymphatic function (Da Mesquita et al. 2018b). In exploring this connection, it was reported that VEGFC-mediated enhancement of meningeal lymphatics in aged mice significantly ameliorated the reduction of exploratory behavior upon exposure to peripheral IL-1β (Goldman et al. 2022). This observation is likely due to the functional link between meningeal lymphatics and the glymphatic system (Da Mesquita et al. 2018a,b) that influences the accessibility of neuromodulators in the CSF to the parenchyma. It is important to note that VEGFR3 is also expressed on approximately 20% of brain blood endothelial cells; therefore, these results should be taken with caution. The use of alternative approaches to study meningeal lymphatic dysfunction has demonstrated that indeed meningeal lymphatics contribute to neurological function and behavior during inflammation. Future investigation should aim to explore the role of meningeal lymphatics in shaping neurological function in physiology, as meningeal-derived cytokines have been implicated in this process.

## VERTEBRAL MENINGEAL LYMPHATICS AND RELATED PATHOLOGIES

The spinal cord, like the brain, is also enclosed by the meninges and a network of bona fide LVs (Antila et al. 2017, Jacob et al. 2019, Louveau et al. 2018). The vertebral lymphatics form a segmented pattern in the epidural space where the dorsal LVs adopt a more connected, circular pattern while the ventral LVs are not connected at the midline (Antila et al. 2017). Whole mount dissection of the meninges from the vertebral bone revealed that dural LVs were around nerve roots (Jacob et al. 2019, Louveau et al. 2018), exiting alongside the spinal nerves and connecting with peripheral lymphatics (Antila et al. 2017, Jacob et al. 2019, Miura et al. 1998). The unique anatomical location of the spinal cord meningeal LVs hints at the bidirectional cross talk between the vessels and peripheral nervous system (Jacob et al. 2019) that can be harnessed to control lymph flow or dorsal root ganglia (DRG) activity.

Vertebral lymphatics can drain macromolecules (Jacob et al. 2019, Ma et al. 2019a, Miura et al. 1998). Tracers injected on one side of the rodent thoracolumbar spinal cord localized to the epidural and dural spaces, and they were detected in the lumen of the ipsilateral vertebral LVs (Jacob et al. 2019). This was observed in mice receiving low-volume tracer by intracerebroventricular or intracisternal injections, reflecting physiological conditions and imaged through noninvasive methods. Injected tracers spread caudally, colocalized with Prox1^+^ vertebral LVs, and outflowed to sacral and iliac lymph nodes (Ma et al. 2019a). These results are in agreement with earlier works demonstrating the drainage of CSF-derived particles through lymphatics originating from the spinal meninges (Brierley & Field 1948, Miura et al. 1998, Zenker et al. 1994).

Spinal cord injury incites cellular damage, and it is mitigated by a local and systemic immune response (Greenhalgh et al. 2020, Salvador & Kipnis 2022). While the response in the spinal cord parenchyma is well studied, recent works have turned their attention to the spinal cord meninges, which are also impacted by the injury (Jacob et al. 2019, Salvador et al. 2023). Contusion injury leads to immune cell accumulation in the spinal cord meninges as well as in the parenchyma (Cohen et al. 2020, Salvador et al. 2023) and, in some cases, is reminiscent of tertiary lymphoid-like structures (TLSs) described in autoimmunity, cancer, and other chronic inflammatory conditions (Aloisi & Pujol-Borrell 2006). The stimuli driving the initiation and maintenance of TLSs remains unresolved (Sato et al. 2023), but molecular cues critical for their organization such as lymphotoxins and chemokines (CXCL13, CCL21, CCL19) were upregulated in the injured spinal cord meninges (Cohen et al. 2020). Aside from contusion injury, spinal cord meningeal TLSs were detected in mouse models of autoimmunity (i.e., EAE) and chronic neurodegeneration (i.e., SOD1 mice) (Cohen et al. 2020). Continuous local antigen stimulation and defective lymphatic drainage may trigger TLS formation (Thaunat et al. 2006). Manipulating vertebral LVs to improve flow and subsequently TLS formation, which may be detrimental to spinal cord pathologies, may be a promising therapeutic strategy.

Aside from immune cell infiltration, *Vegfc* upregulation was detected in the spinal cord meninges after contusion injury, translating to lymphangiogenesis underneath the injury site (Salvador et al. 2023). Notably, this morphological change occurs after laminectomy, which may injure the meninges, and it appears to be mediated by meningeal macrophages (Salvador et al. 2023). Single-cell RNA sequencing and prediction of ligand-receptor interactions also indicate that myeloid cells in the spinal cord parenchyma express lymphangiogenic factors after injury and potentially release them to the CSF to signal to LECs in the meninges. Demyelinated lesions caused by the injection of lysophosphatidylcholine (LPC) also induced focal LV growth that extended to the vertebral bone (Jacob et al. 2019). Augmenting vertebral LV diameter with AAV-VEGFC pretreatment significantly increased the lesion size after LPC injury. This correlated with an increase in leukocyte numbers, suggesting that vertebral LVs facilitate the entry of immune cells to the lesion, which may amplify tissue damage (Jacob et al. 2019). Conversely, interfering with VEGFR3 signaling prevented the expansion of vertebral LV diameter after LPC injury, but it did not significantly reduce the lesion area compared to controls. These suggest that vertebral LVs respond to VEGFC during injury conditions, but other factors may be critical in regulating immune cell infiltration and lesion repair in this context (Jacob et al. 2019).

Deep single-cell RNA sequencing also revealed the transcriptional response of spinal cord meningeal LECs after contusion injury (Salvador et al. 2023). Contusion injury elicited a transcriptional program involving processes related to extracellular matrix organization, response to wounding, and regulation of angiogenesis (Salvador et al. 2023), independent of age. Aged, injured LECs in the spinal cord meninges, however, significantly upregulated genes corresponding to the response to temperature stimulus and sensory perception of pain compared to young, injured LECs. This surprising finding highlights the physical interaction between spinal cord LECs and the DRG and may lead to the discovery of new pain management strategies.

## CONCLUSIONS AND OUTSTANDING QUESTIONS

**Figure 2:**
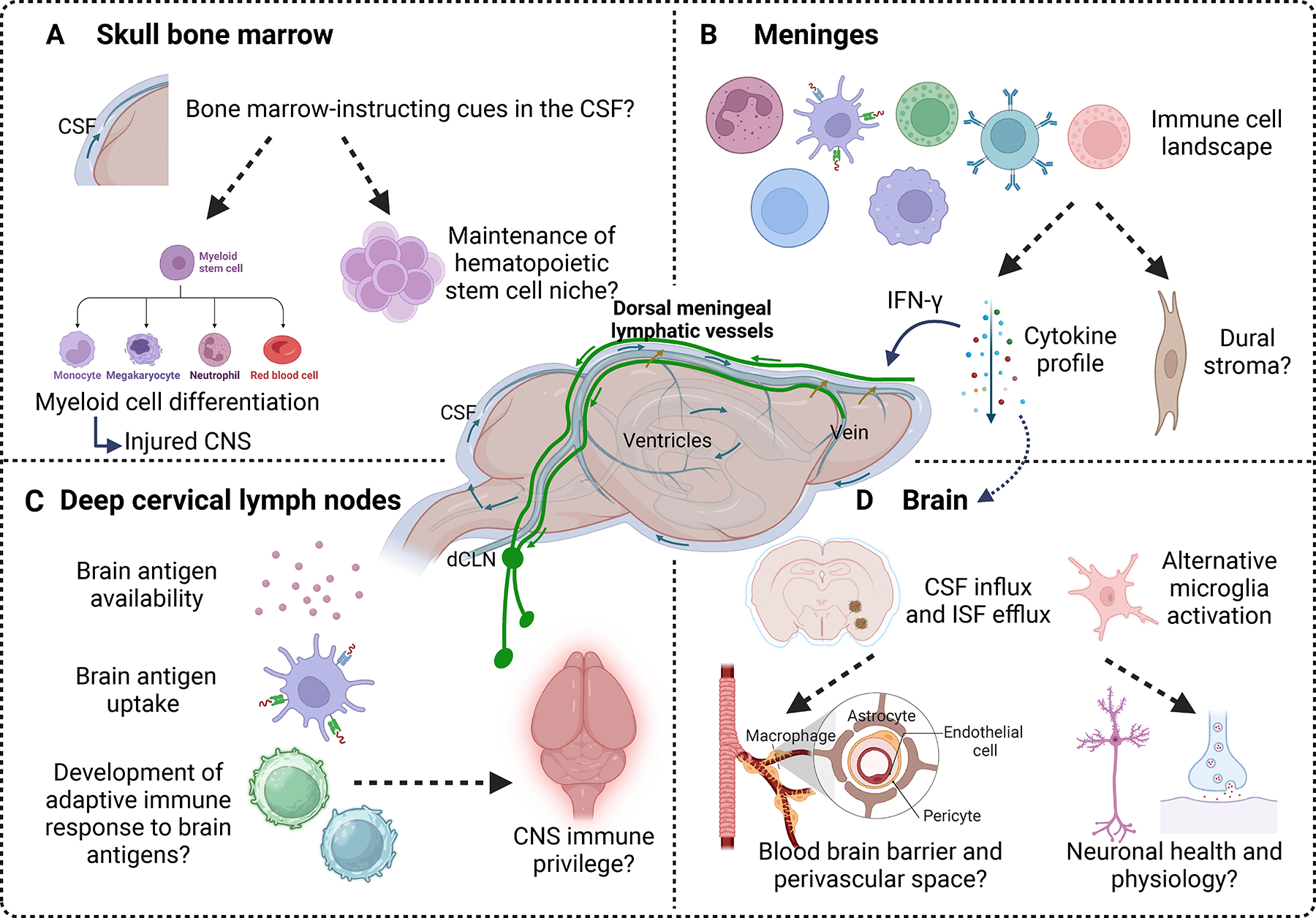
Cranial meningeal lymphatic functions and predicted interactions with brain border compartments. Meningeal lymphatic vessels modulate the drainage of macromolecules and cells into deep cervical lymph nodes. They can also regulate cerebrospinal fluid (CSF) perfusion and interstitial fluid (ISF) efflux in the brain parenchyma. (*a*) Meningeal lymphatics may alter components of the CSF that instruct the skull bone marrow. This feature may affect myeloid cell differentiation in response to central nervous system (CNS) injury or the maintenance of the hematopoietic stem cell niche. (*b*) Changes in meningeal lymphatics can profoundly alter the meningeal immune cell landscape, potentially modifying the cytokine milieu that can affect brain physiology or impair meningeal lymphatic vessels through interferon gamma (IFN-γ) signaling. Alterations in meningeal immunity may also influence the nonhematopoietic compartment of the meninges. (*c*) Manipulation of meningeal lymphatic vessels can impact the delivery and processing of brain-derived antigens in deep cervical lymph nodes (dCLNs), which can shape the development of the local adaptive immune response. (*d*) Meningeal lymphatics can influence CSF/ISF flow in the brain parenchyma and microglial activation. These reported changes can influence the integrity of the blood-brain barrier and neuronal physiology, which can then promote changes in cognitive performance. Solid arrows represent information that has been reported in publications, whereas dashed arrows show that further investigation is required. Figure adapted from images created with Biorender.com. Center meninges/brain image adapted with permission from Louveau et al. (2016).

The excitement accompanying the characterization of meningeal lymphatics led to a redefinition of the mechanisms underlying CNS immune uniqueness. The integrity of the meningeal lymphatic network is critical for CNS waste clearance, in part because of its functional link to the glymphatic system (Da Mesquita et al. 2018b). However, the exact molecular signals and cell types governing this interaction have not been uncovered. Additionally, the consequences of perturbing the glymphatic system on meningeal lymphatics have not been assessed. Aside from the paravascular pathway, the ability of the blood-brain barrier to remove waste from the brain is well recognized (Daneman & Prat 2015), which may also be linked to meningeal lymphatics. In support of this idea, transcriptomic analysis of brain-derived blood endothelial cells revealed significant gene expression changes upon meningeal lymphatic impairment (Da Mesquita et al. 2021b). Because meningeal lymphatics do not exist in isolation, understanding their interaction with other clearance routes and CNS-resident cells, such as astrocytes and microglia, will allow us to decipher their contribution to waste clearance and define therapies with maximal benefit to the CNS (Figure 2).

New research has identified the unanticipated presence of lymphatics in the bone marrow, which are posited to support hematopoiesis and bone regeneration after injury (Biswas et al. 2023). Are bone marrow lymphatics anatomically connected to meningeal lymphatics? Do they display a similar developmental trajectory and dependence on VEGFC for development? Given this finding and the unique ability of CNS-associated bone marrow to deploy tailored immune cells to the CNS (Brioschi et al. 2021, Cugurra et al. 2021, Mazzitelli et al. 2022), it would be of great interest to study how the lymphatics in the bone marrow cooperate with meningeal lymphatics to respond to CNS perturbations. It has also been recently shown that progenitors from the skull bone marrow contribute to VEGFC expression, maintenance of ICP, and skull morphology in the developmental disorder craniosynostosis (Ma et al. 2023). How skull resident cells produce and deliver factors critical for the development of bone marrow and meningeal lymphatics is an open question.

Aging leads to the perturbation of peripheral (Kataru et al. 2022, Nagai et al. 2011) and meningeal (Ahn et al. 2019; Da Mesquita et al. 2018b, 2021b; Rustenhoven et al. 2023) LVs. Aged dorsal meningeal lymphatics display regression and reduced coverage (Da Mesquita et al. 2018b), while basal lymphatics appear more hyperplastic (Ahn et al. 2019). The structural changes may contribute to a reduction in CSF outflow and glymphatic function (Ahn et al. 2019, Louveau et al. 2018, Ma et al. 2017), profoundly impacting neurological disease development (Da Mesquita et al. 2021a). Mechanisms for age-related meningeal lymphatic decline, however, have been understudied. Recent work demonstrated that changes in meningeal immunity during aging underlie lymphatic dysfunction (Rustenhoven et al. 2023). Single-cell RNA sequencing of aged LECs revealed a strong induction of pathways relating to interferon gamma (IFN-γ) response, coupled with a significant expansion of CD4^+^ and CD8^+^ T cells and an increase of IFN-γ expression in the aged meninges (Rustenhoven et al. 2021, 2023). Functionally, exposure of human LECs to IFN-γ disrupted junctional integrity, and IFN-γ overexpression in young dural meninges reduced lymphatic outflow and tracer influx to the parenchyma, mimicking the impairment of clearance observed in aging (Da Mesquita et al. 2018b). Meanwhile, systemic neutralization of IFN-γ in aged mice improved lymphatic drainage to dCLNs without altering glymphatic influx (Rustenhoven et al. 2023), presenting a promising avenue to alleviate waste clearance in aging and neurodegeneration. Because IFN-γ neutralization may diminish pathogen clearance, future work should aim to define alternative targets. In the aged skin for instance, there is increased myeloid cell nitrosative stress and perilymphatic antibody deposition (Kataru et al. 2022), potentially clogging skin lymphatics. Interestingly, the aged dura harbors an expanded population of B cells, particularly IgM^+^ and IgG^+^ plasma cells localized along the sagittal sinus (Brioschi et al. 2021) that can promote antibody aggregation near meningeal lymphatics.

Another key question is what drives the alterations in the meningeal immune cell landscape during aging? Meningeal lymphatic ablation leads to meningeal T cell accumulation (Louveau et al. 2018), raising the question of whether lymphatic dysfunction precedes the changes in the frequency of meningeal immune cells. Aging may also render immune cells deficient in egressing the meninges (Da Mesquita et al. 2021a), leading to accumulation and lymphatic dysfunction. Aside from aging, neurodegenerative disorders can also render meningeal lymphatics dysfunctional (Da Mesquita et al. 2018b). Uncovering the factors that contribute to their impairment may allow us to intervene and preserve the function of meningeal lymphatics in aging.

Depending on the context, manipulation of meningeal LVs can lead to beneficial or detrimental outcomes. We can broadly categorize the effect of manipulating lymphatics as modifying either immune cell trafficking or CSF fluid dynamics (Tavares & Louveau 2021). Untangling the contribution of each manipulation necessitates the development of new tools that preferentially augment one effect to develop refined therapies.

Lastly, more sophisticated imaging techniques have allowed us to survey the nature and fitness of meningeal lymphatics in human patients (Absinta et al. 2017, Ding et al. 2021, Eide et al. 2018, Jacob et al. 2022, Ringstad & Eide 2020). These studies have been pivotal in building a better picture of human lymphatic drainage and understanding the conservation of our preclinical studies in mice to humans. Findings from imaging patients may be used as diagnostic tools for progressive neurodegenerative disorders, such as AD and PD, so that timely interventions, possibly through the manipulation of the meningeal lymphatic system, can be given.

In summary, the therapeutic potential of the meningeal lymphatic system is evident, though not yet fully realized. As we continue to probe its intricacies, the development of targeted interventions for neurodegenerative conditions becomes increasingly plausible. Future research should emphasize understanding not only the system’s function but also how it can be harnessed for clinical benefit.

## Supplementary Material

Supplemental Appendix and Supplemental Table 1
