# Supplementary material for: Meningeal Lymphatics in Central Nervous System Diseases
Source: Annu Rev Neurosci. Author manuscript; Available in PMC 2025 Aug 1. (PMC12051392; doi:10.1146/annurev-neuro-113023-103045)
Supplement: Supplemental Appendix and Supplemental Table 1 [file NIHMS2069376-supplement-Supplemental_Appendix_and_Supplemental_Table_1.pdf]

Supplemental Material: Annu. Rev. Neurosci. 2024. 47:323-344
https://doi.org/10.1146/annurev-neuro-113023-103045
Meningeal Lymphatics in Central Nervous System Diseases
Salvador, Abduljawad, and Kipnis

Supplemental Appendix

Manipulating the meningeal lymphatic system

Studies elucidating the molecular cues that regulate the growth and maintenance of peripheral lymphatics have informed the development of multiple approaches to manipulate meningeal LV and enumerate their functions in homeostasis and disease(Oliver et al 2020). Haploinsufficiency of the transcription factor *Prox1*, the master regulator of LEC fate, results in abnormal patterning and leakiness of peripheral lymphatic beds(Harvey et al 2005, Wigle & Oliver 1999). Macromolecules injected into the CSF of *Prox1* heterozygotes have reduced drainage to the cLN and reduced perfusion into the parenchyma compared to controls, suggesting that the integrity of lymphatic vessels is crucial to supporting CNS fluid movement(Da Mesquita et al 2018, Louveau et al 2018).

Signaling of VEGFC or VEGFD to their cognate receptor, VEGFR3(Jeltsch et al 1997, Stacker et al 2002), is a main driver of lymphangiogenesis. Transgenic mice expressing a chimeric soluble "VEGFC/D trap," consisting of the ligand binding portion of VEGFR3 (domains 1-3) fused with the Fc receptor domain of IgG under the keratin 14 promoter (K14-Vegfr3-IgG), displayed regression of skin lymphatics and lymphedema(Mäkinen et al 2001). Despite the keratinocyte-directed expression of the VEGFC/D trap, meningeal LV adjacent to the superior sagittal sinus (SSS) and the pterygopalatine artery (PPA) also atrophied(Aspelund et al 2015). This was accompanied by a reduction of tracer drainage to the dCLN upon intracisternal injection indicating that meningeal LV maintenance is dependent on VEGFC/D signaling. Alternatively, intraperitoneal injection of adeno-associated virus (AAV)-VEGFC/D trap (AAV-mVEGFR3(1-3)-Ig) in newborn (P0) mice was sufficient to induce persistent atrophy of cranial and spinal cord

Downloaded from www.annualreviews.org. Guest (guest) IP: 128.252.154.7 On: Tue, 15 Apr 2025 18:08:36

Downloaded from www.annualreviews.org. Guest (guest) IP: 128.252.154.7 On: Tue, 15 Apr 2025 18:08:36

meningeal LV compared to controls (AAV-mVEGFR3(4-7)-Ig) without compromising blood vessel growth(Antila et al 2017). Treatment of adult mice also promoted regression of dorsal meningeal LV and impaired drainage, but only partial regression of spinal cord meningeal LV(Li et al 2023), suggesting different requirement of cranial and spinal cord meningeal lymphatics to VEGFR3 signaling.

Among the VEGFR3 ligands, VEGFC was found to be indispensable for meningeal LV development. Mice heterozygous for VEGFC ($Vegfc^{LacZ/+}$) lack cranial and spinal meningeal LV in pups and in adult mice, but VEGFD-deficient mice retained an intact meningeal LV network(Antila et al 2017). Conditional depletion of *Vegfc* ($Rosa26CreER^{T2};Vegfc^{flox/flox}$) starting from either P3 or P8 halted meningeal LV development, and it was instead replaced by LYVE1+CD206+F480+ cells(Antila et al 2017). Moreover, mice with hypomorphic *Vegfc* alleles illustrated the critical VEGFC dosage for supporting normal meningeal LV growth(Antila et al 2017).

Conversely, global depletion of VEGFR3 receptor ($Rosa26\text{-}CreER^{T2};Vegfr3^{flox/flox}$)(Antila et al 2017) and conditional deletion of VEGFR3 in Prox1+ cells ($Prox1\text{-}CreER^{T2};Vegfr3^{flox/flox}$)(Ahn et al 2019, Li et al 2023) in adult mice led to significant regression of dorsal meningeal LV, the basal meningeal LV near the sigmoid and petrosquamosal sinuses, and meningeal LV around the spinal cord(Li et al 2023), and an impairment of CSF drainage to the dCLN. In adult mice, intraperitoneal administration of blocking antibodies to VEGFR3 (clone mF4-31C1) designed to antagonize its binding to VEGFC(Pytowski et al 2005) also resulted in the regression of dorsal meningeal LV and impaired lymphatic drainage(Li et al 2023). However, meningeal LV in the base of the skull and around the spinal cord were not impacted, indicating

Downloaded from www.annualreviews.org. Guest (guest) IP: 128.252.154.7 On: Tue, 15 Apr 2025 18:08:36

that dorsal meningeal LV have greater susceptibility to perturbations to VEGFR3 signaling compared to other lymphatic beds in the CNS(Ahn et al 2019, Antila et al 2017, Li et al 2023).

Intraperitoneal injection of sunitinib, a tyrosine kinase inhibitor that inhibits signaling downstream of the VEGFC-VEGFR3 pathway, also resulted in significant regression of dorsal meningeal lymphatics(Antila et al 2017). Sunitinib treatment for 2 weeks led to meningeal LV atrophy, especially in the SSS region, but its withdrawal allowed partial regrowth. Though the function of the newly grown vessels was not assessed, these findings illustrate the incredible plasticity of cranial meningeal LV. Collectively, the studies indicate the dependence of meningeal LV maintenance on continuous VEGFR3 signaling. Besides VEGFR3 signaling, phospholipase C-γ (PLC-γ) together with components of the CLEC2-PDPN signaling axis have been implicated in LV formation and the regulation of lymph flow(Finney et al 2012, Sweet et al 2015). Global PLC-γ deficiency can cause the improper partitioning of LV and blood vessels(Ichise et al 2009), and reduced lymph flow in the gastrointestinal tract (Bálint et al 2019). Meningeal LV maturation and drainage of molecules to the dCLN were also impaired(Bálint et al 2019), implying that other factors also contribute to meningeal LV development.

Aside from interfering with lymphangiogenic signaling, meningeal LV can also be ablated with the photodynamic compound verteporfin, or Visudyne®(Da Mesquita et al 2018, Louveau et al 2018). Illuminating the compound with a nonthermal laser leads to the fragmentation of peripheral lymphatics through the release of reactive oxygen species (Tammela et al 2011). It can also be activated through the skull after injection into the CSF, resulting in significant reduction of meningeal lymphatic coverage and drainage capacity to the dCLN, and increased accumulation of endogenous T cells in the meninges(Louveau et al 2018). This also preserves the integrity of blood vasculature, the glia limitans, and ventricular volume(Da Mesquita et al 2018, Louveau et al 2018).

Downloaded from www.annualreviews.org. Guest (guest) IP: 128.252.154.7 On: Tue, 15 Apr 2025 18:08:36

Meningeal lymphatic drainage function can also be impaired using surgical approaches(Louveau et al 2015). Surgical ligation(Da Mesquita et al 2018, Louveau et al 2018) or cauterization(Song et al 2020) of afferent lymphatics to dCLN was sufficient to reduce the drainage of macromolecules and cells injected to the CSF(Da Mesquita et al 2018, Louveau et al 2018, Rustenhoven et al 2021) without altering lymph node size. Both meningeal lymphatic photodynamic ablation and ligation reduced the perfusion and efflux of CSF tracers into the parenchyma(Da Mesquita et al 2018).

Given the importance of VEGFC-VEGFR3 signaling in meningeal LV growth and maintenance, VEGFC supplementation offers a viable strategy to enhance lymphangiogenesis. Mice that received intracisternal injection of recombinant VEGFC displayed an increase in meningeal LV diameter when measured 7 days post-injection(Louveau et al 2015). Slow, transcranial delivery of hydrogel-encapsulated VEGFC that specifically binds to VEGFR3 also enhanced meningeal LV diameter, without altering blood vasculature coverage(Da Mesquita et al 2018). Adeno-associated virus (AAV1 or AAV9)(Antila et al 2017, Da Mesquita et al 2018, Da Mesquita et al 2021, Song et al 2020) or mRNA delivery vectors(Song et al 2020) allowing the ectopic expression of VEGFC have also been used to facilitate a more sustained delivery of the lymphangiogenic factor. After delivery to the CSF, AAV9-, AAV1-CMV-VEGFC, or VEGFC mRNA induced an increase in VEGFC levels in the CSF and meningeal tissue(Song et al 2020) together with an increase in LV diameter(Da Mesquita et al 2018, Song et al 2020). However, drainage of molecules to the dCLN was not significantly enhanced after delivery of AAV1-CMV-VEGFC to young-adult mice, perhaps implying a ceiling effect of their drainage capacity(Da Mesquita et al 2018).

While the methods outlined above have been effective in targeting meningeal lymphatics, they have inherent limitations which can be fine-tuned to increase specificity through advances in

transcriptomics(Rustenhoven et al 2023, Salvador et al 2023). Nonetheless, they have been critical in ascertaining the role of meningeal lymphatics in multiple neurological disorders discussed in the following sections (Figure 1).

Suppl References

Ahn JH, Cho H, Kim J-H, Kim SH, Ham J-S, et al. 2019. Meningeal lymphatic vessels at the skull base drain cerebrospinal fluid. *Nature* 572: 62-66

Antila S, Karaman S, Nurmi H, Airavaara M, Voutilainen MH, et al. 2017. Development and plasticity of meningeal lymphatic vessels. *J Exp Med* 214: 3645-67

Aspelund A, Antila S, Proulx ST, Karlsen TV, Karaman S, et al. 2015. A dural lymphatic vascular system that drains brain interstitial fluid and macromolecules. *J Exp Med* 212: 991-99

Bálint L, Ocskay Z, Deák BA, Aradi P, Jakus Z. 2019. Lymph flow induces the postnatal formation of mature and functional meningeal lymphatic vessels. *Front Immunol* 10: 3043

Da Mesquita S, Louveau A, Vaccari A, Smirnov I, Cornelison RC, et al. 2018. Functional aspects of meningeal lymphatics in ageing and Alzheimer's disease. *Nature* 560: 185-91

Da Mesquita S, Papadopoulos Z, Dykstra T, Brase L, Farias FG, et al. 2021. Meningeal lymphatics affect microglia responses and anti-Aβ immunotherapy. *Nature* 593: 255-60

Finney BA, Schweighoffer E, Navarro-Núñez L, Bénézech C, Barone F, et al. 2012. CLEC-2 and Syk in the megakaryocytic/platelet lineage are essential for development. *Blood* 119: 1747-56

Harvey NL, Srinivasan RS, Dillard ME, Johnson NC, Witte MH, et al. 2005. Lymphatic vascular defects promoted by Prox1 haploinsufficiency cause adult-onset obesity. *Nat Genet* 37: 1072-81

Ichise H, Ichise T, Ohtani O, Yoshida N. 2009. Phospholipase Cgamma2 is necessary for separation of blood and lymphatic vasculature in mice. *Development* 136: 191-95

Jeltsch M, Kaipainen A, Joukov V, Meng X, Lakso M, et al. 1997. Hyperplasia of lymphatic vessels in VEGF-C transgenic mice. *Science* 276: 1423-25

Li Z, Antila S, Nurmi H, Chilov D, Korhonen EA, et al. 2023. Blockade of VEGFR3 signaling leads to functional impairment of dural lymphatic vessels without affecting autoimmune neuroinflammation. *Sci Immunol* 8: eabq0375

Louveau A, Herz J, Alme MN, Salvador AF, Dong MQ, et al. 2018. CNS lymphatic drainage and neuroinflammation are regulated by meningeal lymphatic vasculature. *Nat Neurosci* 21: 1380-91

Louveau A, Smirnov I, Keyes TJ, Eccles JD, Rouhani SJ, et al. 2015. Structural and functional features of central nervous system lymphatic vessels. *Nature* 523: 337-41

Mäkinen T, Jussila L, Veikkola T, Karpanen T, Kettunen MI, et al. 2001. Inhibition of lymphangiogenesis with resulting lymphedema in transgenic mice expressing soluble VEGF receptor-3. *Nat Med* 7: 199-205

Oliver G, Kipnis J, Randolph GJ, Harvey NL. 2020. The lymphatic vasculature in the 21st century: novel functional roles in homeostasis and disease. *Cell* 182: 270-96

Downloaded from www.annualreviews.org. Guest (guest) IP: 128.252.154.7 On: Tue, 15 Apr 2025 18:08:36

Pytowski B, Goldman J, Persaud K, Wu Y, Witte L, et al. 2005. Complete and specific inhibition of adult lymphatic regeneration by a novel VEGFR-3 neutralizing antibody. *J Natl Cancer Inst* 97: 14-21
Rustenhoven J, Drieu A, Mamuladze T, de Lima KA, Dykstra T, et al. 2021. Functional characterization of the dural sinuses as a neuroimmune interface. *Cell* 184: 1000-16.e27
Rustenhoven J, Pavlou G, Storck SE, Dykstra T, Du S, et al. 2023. Age-related alterations in meningeal immunity drive impaired CNS lymphatic drainage. *J Exp Med* 220
Salvador AFM, Dykstra T, Rustenhoven J, Gao W, Blackburn SM, et al. 2023. Age-dependent immune and lymphatic responses after spinal cord injury. *Neuron*
Song E, Mao T, Dong H, Boisserand LSB, Antila S, et al. 2020. VEGF-C-driven lymphatic drainage enables immunosurveillance of brain tumours. *Nature* 577: 689-94
Stacker SA, Achen MG, Jussila L, Baldwin ME, Alitalo K. 2002. Lymphangiogenesis and cancer metastasis. *Nat Rev Cancer* 2: 573-83
Sweet DT, Jiménez JM, Chang J, Hess PR, Mericko-Ishizuka P, et al. 2015. Lymph flow regulates collecting lymphatic vessel maturation in vivo. *J Clin Invest*
Tammela T, Saaristo A, Holopainen T, Ylä-Herttuala S, Andersson LC, et al. 2011. Photodynamic ablation of lymphatic vessels and intralymphatic cancer cells prevents metastasis. *Sci Transl Med* 3: 69ra11
Wigle JT, Oliver G. 1999. Prox1 function is required for the development of the murine lymphatic system. *Cell* 98: 769-78

Downloaded from www.annualreviews.org. Guest (guest) IP: 128.252.154.7 On: Tue, 15 Apr 2025 18:08:36

Supplemental Table 1. Summary of methods to manipulate meningeal lymphatic vessels and their applications.

Method/mouse line/virus	Specificity	Effects	Applications	References
Prox1^{het} mice	Global deficiency of Prox1	Reduction of intracisternal-injected tracer and T cell drainage into the dCLN; increase in endogenous T cell numbers in the meninges; reduced CSF influx to the brain		(Louveau et al 2018)
VEGFC/D trap mice (i.e., K14-Vegfr3-IgG)	Keratinocyte-specific expression of soluble VEGFR3	Atrophy of meningeal lymphatic vessels; reduced tracer drainage to the dCLN (Aspelund et al 2015)	AD – increased brain retention of injected tau	(Patel et al 2019)
			TBI – reduction of T cell infiltration around lesion	(Wojciechowski et al 2020)
Vegfc$^{LacZ/+}$ mice	Global and constitutive	Absence of cranial and spinal		(Antila et al 2017)

		depletion of VEGFC	meningeal lymphatic vessels in pups and adulthood		
Rosa26CreER^{T2}*;Vegfc*$^{flox/flox}$ mice		Conditional depletion of VEGFC (from P3 or P8)	Interruption of meningeal LV development replaced by macrophage-like cells		(Antila et al 2017)
Rosa26CreER^{T2}*;Vegfr3*$^{flox/flox}$ mice		Global, postnatal depletion of VEGFR3	Absence of cranial and spinal meningeal LV		(Antila et al 2017)
Prox1-CreER^{T2};*Vegfr3*$^{flox/flox}$ mice		Conditional deletion of VEGFR3 in Prox1^{+} cells	Regression of cranial (dorsal and basal) and spinal meningeal LV	EAE – does not alter disease progression (Li et al 2023)	(Ahn et al 2019, Li et al 2023)
Plc2γ$^{-/-}$ mice		Global deficiency of PLC-γ	Reduction of dorsal meningeal LV coverage		(Bálint et al 2019)
Overexpression of soluble VEGFR3 (AAV-VEGFC/D trap)		Intraperitoneal delivery in newborn mice	Atrophy of cranial and spinal cord meningeal LV		(Antila et al 2017)
		Intraperitoneal delivery in adult mice	Atrophy of dorsal, cranial meningeal LV but partial effect on basal and spinal meningeal LV	EAE – does not alter disease progression	(Li et al 2023)
		Intracisternal delivery at P7	Atrophy of dorsal cranial meningeal LV		(Antila et al 2017)
Anti-VEGFR3 blocking antibodies (mF4-31C1)		Intraperitoneal delivery in adult mice	Atrophy of dorsal cranial meningeal LV	EAE – does not alter disease progression	(Li et al 2023)
Pharmacological inhibition of receptor tyrosine kinase	Sunitinib	Intraperitoneal delivery in adult mice	Reversible regression of dorsal cranial meningeal LV		(Antila et al 2017)

Downloaded from www.annualreviews.org. Guest (guest) IP: 128.252.154.7 On: Tue, 15 Apr 2025 18:08:36

Downloaded from www.annualreviews.org. Guest (guest) IP: 128.252.154.7 On: Tue, 15 Apr 2025 18:08:36

	MAZ51	Intraperitoneal delivery in adult mice	Reduction of lymphangiogenesis in healthy and diseased dorsal meninges, and diseased cribriform plate	EAE – reduction of clinical score	(Hsu et al 2019)
Photodynamic ablation using Visudyne®		Intracisternal delivery and illumination in adult mice	Reduction of dorsal meningeal LV coverage; reduction of intracisternal delivered tracer and cells to the dCLN; reduced CSF influx to the brain parenchyma	EAE – reduction of disease severity	(Louveau et al 2018)
				AD – worsening of amyloid burden; impairment of anti-Aβ immunotherapy	(Da Mesquita et al 2018b, Da Mesquita et al 2021b)
				TBI – increase in gliosis and cognitive decline	(Bolte et al 2020)
				Brain tumors – impairment of anti-tumor immune responses	(Hu et al 2020, Zhou et al 2022)
				Intracerebral hemorrhage – reduction of hematoma clearance	(Tsai et al 2022)
				Neurotropic viral infection – increase in mortality and viral burden	(Li et al 2022)
				Sickness behavior – reduced exploratory activity; alteration of microglial activation	(Goldman et al 2022)
Surgical ligation of lymphatic vessels		Afferents to the dCLN	Reduction of intracisternal delivered tracer	EAE – delay of disease progression	(Louveau et al 2018)

Downloaded from www.annualreviews.org. Guest (guest) IP: 128.252.154.7 On: Tue, 15 Apr 2025 18:08:36

		and cell drainage to the dCLN; reduced CSF influx to the brain parenchyma	AD – exacerbation of Aβ pathology	(Wang et al 2019)
			PD – worsening of α-synuclein aggregation	(Ding et al 2021, Zou et al 2019)
			Intracerebral hemorrhage – reduction of hematoma clearance	(Tsai et al 2022)
			Neurotropic viral infection – increase in mortality and viral burden	(Li et al 2022)
Ectopic VEGFC overexpression	Intracisternal delivery of AAV1-; AAV9-; VEGFC mRNA; or VEGF protein(Louveau et al 2015); Transcranial delivery of VEGFC protein with hydrogel	Increase in cranial meningeal LV diameter	Aging – increased CSF drainage to dCLN and perfusion to brain parenchyma; improved cognition	(Da Mesquita et al 2018b)
			AD – reduction of Aβ pathology dependent on model and time of treatment; improved Aβ clearance upon immunotherapy of aged mice	(Da Mesquita et al 2021b, Wen et al 2018)
			Brain tumors – improved anti-brain tumor immune response	(Hu et al 2020, Song et al 2020, Zhou et al 2022)
			Intracerebral hemorrhage – reduction of hematoma volume and debris clearance	(Tsai et al 2022)

Downloaded from www.annualreviews.org. Guest (guest) IP: 128.252.154.7 On: Tue, 15 Apr 2025 18:08:36

			TBI – Reduction of gliosis in aged animals; reduction of brain edema	(Bolte et al 2020, Liao et al 2023)
			Sickness behavior – increased locomotion of aged, sick animals	(Goldman et al 2022)
			Craniosynostosis – Normalization of ICP, CSF perfusion, cognition	(Ma et al 2023)
	Intralumbosacral injection of AAV9-VEGFC	Increase in spinal LV diameter	Demyelination – increase in lesion size and immune cell infiltrates	(Jacob et al 2019)

Downloaded from www.annualreviews.org. Guest (guest) IP: 128.252.154.7 On: Tue, 15 Apr 2025 18:08:36
